# Occupation exposed to road-traffic emissions and respiratory health among Congolese transit workers, particularly bus conductors, in Kinshasa: a cross-sectional study

**DOI:** 10.1186/s12199-017-0608-9

**Published:** 2017-03-20

**Authors:** Etongola Papy Mbelambela, Ryoji Hirota, Masamitsu Eitoku, Sifa Marie Joelle Muchanga, Hidenori Kiyosawa, Kahoko Yasumitsu-Lovell, Ontshick Leader Lawanga, Narufumi Suganuma

**Affiliations:** 1 Department of Environmental Medicine, Kochi University Medical School, Oko-cho Kohasu, Nankoku, Kochi 783-8505 Japan; 2 Department of Gynecology and Obstetrics, University of Kinshasa, Kinshasa, Democratic Republic of the Congo; 3 Gillberg Neuropsychiatry Centre, University of Gothenburg, Gothenburg, Sweden; 4 Department of Mathematic, University of Kinshasa, Kinshasa, Democratic Republic of the Congo

**Keywords:** Bus conductors, Respiratory health, Road traffic emissions, Transit workers

## Abstract

**Objectives:**

Road-traffic emissions (RTE) induce adverse health effects, notably respiratory symptoms and respiratory diseases, as a result of pollutants deposited into the respiratory tract. The aim of this study was to evaluate the association between occupation groups of Congolese transit workers exposed to RTE, particularly bus conductors and respiratory health, in Kinshasa.

**Methods:**

A cross-sectional study was conducted from 2015 April 20^th^ to May 14^th^, whose participants were bus conductors (*n* = 110), bus drivers (*n* = 107), taxi-motorcyclists (*n* = 102) and high school teachers (control group; *n* = 106). Subjects had completed the American Thoracic Society respiratory symptom questionnaire. Lung function test was performed by spirometry. Air pollutants levels of PM_2.5_, NO_2_ and SO_2_ were measured between 7:30 and 8:30 and 16:30–17:30 using a portable gas monitor.

Multivariate analysis was performed to evaluate the association between occupation exposed to RTE and impaired pulmonary function, after adjustment by plausible confounders.

**Results:**

The prevalence of mixed syndrome was 21.9% for bus conductors, 10.9% for bus drivers, 15.4% for taxi-motorcyclists and 7.1% for high school teachers with (*p* < 0.05). The risk of developing a mixed syndrome was seven times higher among bus conductors [OR = 7.64; 95% CI: 1.83–31.67; *p* < 0.05] than other groups. Additionally, the prevalence of respiratory syndromes increased with the duration of exposure.

**Conclusions:**

Occupation exposed to RTE is associated with impaired pulmonary function and the prevalence of respiratory symptoms among transit workers, especially bus conductors. Furthermore, this association increases with the duration of exposure suggesting the necessity to regulate these categories of occupations and to apply preventives measures.

## Introduction

In the coming decades, road transport is likely to remain a significant contributor to air pollution in most of cities. Many urban trips cover distances shorter than 6 km and the average traffic emissions per driving distance are very high in urban areas due to the low effectiveness of catalytic converters in the initial minutes of engine operation. Also, poorly maintained vehicles that lack exhaust after treatment systems are responsible for a major part of pollutant emissions [1]. Preliminary assessments indicate that, each year, ten thousands persons are affected by diseases related to road-traffic emissions in the European region [2].

Road-Traffic Emissions (RTE) has been associated to different sources. Among them, exhaust pipe emissions, contributions from friction processes and resuspended road dust are known to have adverse health effects, such as throat pain, phlegm, chronic rhinitis and chronic pharyngitis among bus drivers, bus conductors and taxi drivers [3, 4]. The pollutants of greatest concern, due to their impact over health, are particulate matter (PM), ground-level ozone (O_3_), NO_2_, carbon monoxide (CO) and volatile organic compounds (VOC). One of the most important sectors that produce these pollutants is related to the transportation sector [5, 6].

In developed countries, some studies related to exposure to air pollution and adverse health outcomes have shown an increase of decease for stomach cancer, lung cancer, bronchitis, emphysema and asthma. In addition, Buckeridge et al., in the Toronto study had shown a high prevalence of bronchitis, pneumonia, chronic obstructive pulmonary disease and hospital admissions for exhibited individuals [7]. Furthermore, the exposure to traffic-related pollutants such as diesel exhaust particles (DEP) or NO_2_ may contribute to increased respiratory morbidity among adolescents, urban residents and asthmatics. Also, this exposure has been correlated to an increased risk of chronic obstructive pulmonary diseases among railroad workers exposed [8, 9].

**Fig. 1:**
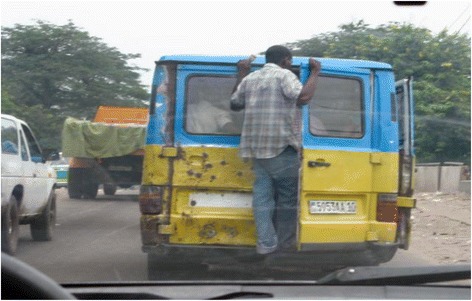
Bus conductors outside of micro bus during the round in Kinshasa, Democratic Republic of the Congo

A long-term exposure to traffic and PM_2.5_ at relatively low levels concentration (μg/m^3^) were reported to be associated with lower forced expiratory volume in first second (FEV_1_) and forced vital capacity (FVC). In addition, a marked decrease in lung function rate and a high blood pressure were observed in the Veterans Administration Normative Aging Study [10, 11]. Research conducted in the Sub-Saharan African countries regarding the effects of air pollutants among traffic workers and non-traffic workers also showed similar health outcomes [3, 4, 12]. However, the lack of studies related to occupation groups of transit workers exposed to RTE and respiratory health in the Democratic Republic of Congo (DRC), remains a matter of making the comparative results with previous studies. Actually, transit workers group of bus conductors constitutes one of vulnerable population. Their vulnerability is mainly due to the exhaust pipe emissions inhaled when calling, persuading potential clients and their outside position during the round (Fig. 1). Majority of them consists of young people, with low education level, low socio-economic status and without health insurance coverage.

No official or national records about socio-economic profiles, literacy rates and educational level have been taken in this population. The aim of this study was to evaluate the association between occupation groups of Congolese transit workers exposed to RTE, particularly bus conductors and the respiratory health, in Kinshasa, the capital of DRC.

## Materials and methods

### Study design and participants

A cross-sectional study was conducted in Kinshasa, DRC, from 2015 April 20^th^ to May 14^th^. A total of 517 subjects, including 392 RTE-exposed transit workers (138 bus conductors, 138 bus drivers, 116 taxi motorcyclists) and 125 high school teachers (control group) were enrolled in this study. Among the enrolled, 31 subjects did not meet the required conditions for spirometry performance, 33 spirometry items were unacceptable, and 28 questionnaires had a missing data. Finally 425 subjects were eligible for this study, including, 110 bus conductors, 107 bus drivers, 102 taxi motorcyclists and 106 high school teachers.

The inclusion criteria were: males, between 20 and 40 years old, who have been working at least three years before prior survey. The exclusion criteria were: females, history of active chronic lung disease or lung surgery and under corticosteroid therapy or beta mimetic-medication.

### Ethical considerations and data collection

The study protocol was approved by the Environment Department of the Provincial Government of Kinshasa, DRC and the ethics committee of Kochi University Medical School, Japan (approval number: 2015–007). After a thorough explanation of the study content, a written informed consent was obtained from each participant.

A translated French version of the American Thoracic Society respiratory symptom questionnaire (ATS) was anonymously completed by all participants to assess respiratory health [13]. Given the low literacy of some of the participants, the questionnaire was administered with assistance of surveyors (three medical doctors and one nurse). Information on respiratory symptoms, personal and familial medical history, lifestyle, employment history, socio-economic status, smoking status, alcohol consumption, physical activity and household characteristics were collected.

The anthropometric parameters (height, weight), blood pressure, lung function parameters were assessed.

### Physical examination

Body weight of each subject was measured using the scale Omron HBF-217 (Karada scan, Kyoto Japan) by following instructions from the manufacturer. Body height measurement was assessed using Microtoise Seca 206 (Japan). Body mass index (BMI) was calculated and classified according to WHO guidelines for BMI [14].

Blood pressure measurement was performed by the use of an electronic sphygmomanometer typed Omron HEM-6200 (Japan) following the recommendation of the American Heart Association and Joint National Committee for management of hypertension in adult (JNC-8) [15, 16].

### Lung function testing (spirometry)

Before spirometry performance, surveyors explained the importance and the procedure of the test. Participants were asked to follow these recommendations: no heavy physical exercises 30 min, no corticoid medication 24 h, no cigarette smoking 1 h, no heavy meal 2 h, and no alcohol consumption 4 h before the test.

From 2015 April 20^th^ to May 14^th^ twice a day between 12 h30 and 14 h30 and 4 h30-6 h30 PM for participant units of 15–20 individuals, Forced vital capacity (FVC), forced expiratory volume in first second (FEV_1_) and FEV_1_/FVC ratio was measured using an Autospiro Minato AS-470 (Medical Science Company Ltd, Osaka) spirometry after calibration.

The recommendations of ATS on acceptability and reproducibility of the test were followed including: the duration of exhalation at least 6 s for adults, the FVC and FEV_1_ were measured through a series of three acceptable items free from artifacts in each participant; the effort provided by the subject was reproductive and maximum. The change in lung function (△FVC and △FEV_1_) which is defined as the difference in the lung function between the last observation and the first observation was less than 200 mm [17].

Spirometry measured values of FVC and FEV_1_ were compared with predicted normal values based on the regression equation from National health and nutrition examination survey (NHANES III)/Hankinson et al. 1999 for male American-African [18]:

$$ \mathrm{F}\mathrm{V}\mathrm{C}=-0.1933+{64.10}^{-5}\times \mathrm{age}-{269.10}^{-6}\times \mathrm{age}\times \mathrm{age}+{186.10}^{-5}\times \mathrm{height}\left(\mathrm{cm}\right)\times \mathrm{height}\left(\mathrm{cm}\right) $$

$$ {\mathrm{FEV}}_1=0.5536-{13.10}^{-3}\times \mathrm{age}-{172.10}^{-6}\times \mathrm{age}\times \mathrm{age}+{14.10}^{-5}\times \mathrm{height}\left(\mathrm{cm}\right)\times \mathrm{height}\left(\mathrm{cm}\right) $$

$$ \mathrm{F}\mathrm{E}\mathrm{V}1\%={\mathrm{PredictedFEV}}_1/\mathrm{Predicted}\ \mathrm{F}\mathrm{V}\mathrm{C} $$

The impaired pulmonary function was calculated in accordance with the ATS guidelines, as follows:

1. Obstructive syndrome defined by: FEV_1_/FVC ratio <70% of predicted.
2. Restrictive syndrome defined by: FEV_1_/FVC ratio is normal or increase and FVC < 80% of predicted.
3. Mixed syndrome defined by combination of restrictive and obstructive syndrome abnormalities.

### Ambient Air measurements

Choice of locations is related to geographical, traffic volume and administration distribution of Kinshasa. Location 1 is the road that goes from East to down town with four lanes, fourteen intersections, and fourteen bus stops. Location 2 is the road that goes from western to down town with four lanes, twelve intersections, and eighteen bus stops. Location 3 is the road that goes from south to down town with eight lanes, sixteen intersections, twenty two bus stops and eight bus stations. Location 4 is the road that goes from North to down town with eight lanes, fourteen intersections, sixteen bus stops and nine bus stations.

Outdoor PM_2.5_ (μg/m^3^) was assessed at each location at 50 m around the road intersections using the Digital dust indicator cyclone LD-5, a portable gas monitor (Sibata kagaku, Japan), NO_2_ was measured using the Gas Alert extreme GAXT-D-DL 2313, Single-Gas Detector, (BW Technologies, USA), and Sulfur Dioxide (SO_2_) using Gas Alert extreme GAXT-S-DL 0515, single-Gas Detector (BW Technologies, USA). All measurements were performed between 7 h30 and 8 h30 and 16 h30-17 h30 at four locations with the same instruments. Each value for every location was represented by an average of different intersections in the same location. Air quality guidelines of World Health Organization were used as reference values [19].

To assess the air quality in the bus, a portable monitor gas was placed inside and in front of the bus after closing the windows, doors and stopping the air conditioner. Measurements for high school teachers were taken in the classroom.

Traffic volume measurements were also measured, and meteorological parameters (temperature, wind speed, wind direction, humidity, and precipitation) were provided from the regional meteorological agency of Kinshasa, DRC.

### Statistical analysis

To test the intergroup differences, Chi-square test and analysis of variance (ANOVA) were performed for categorical and numerical variables respectively. The results were summarized in tables and graphics. Continuous variables were represented by mean and standards deviation (mean; SD) and categorical variables were represented by proportion (%).

Univariate analysis was carried out to evaluate the association between different risk factors and impaired pulmonary function. Multivariate analysis was performed to evaluate the association between occupation group exposed to RTE and impaired pulmonary function, after adjustment for age, BMI, working years, physical activity, alcohol consumption, smoking status, and personal medical history. Results were presented as adjusted odds ratio (aOR), confidence interval (CI).

Statistical significance was defined as a 2-sided *P* value less than 0.05. All analyses were performed using STATA Software version 13.0 for windows.

## Results

In total, 425 subjects participated in this study (110 bus conductors, 107 bus drivers, 102 taxi motorcyclists, and 106 teachers of high school). Baseline characteristics of the study population are shown in Table 1. The mean-age was 26.6 (4.2) years for bus conductors, 29.9 (6.2) for bus drivers, 29.2 (7.2) for taxi motorcyclists and 32.3 (5.6) for teachers with *p* < 0.001. 32. 7% of bus conductors, 64.5% of bus drivers, 20.6% of taxi motorcyclists and 30.2% of high school teachers had worked for at least 5 years with *p* < 0.001. Current smokers represented 79% of bus conductors, 71% of bus drivers, 40% of taxi motorcyclists and 24% of high school teachers with *p* < 0.001.

**Table 1:** General characteristics of the study population

Characteristics	Bus conductors *n* = 110	Bus drivers *n* = 107	Taxi motorcyclists *n* = 102	High school teachers *n* = 106	*P* value
Age (year) Mean (SD)	26.6 (4.2)	29.9 (6.2)	29.2 (7.2)	32.3 (5.6)	<0.001^a^
BMI (Kg/m^2^) Mean (SD)	23 (0.8)	23 (2.3)	22.7 (1.0)	23.5 (0.9)	0.002^a^
SBP (mmHg) Mean (SD)	125.6 (10.5)	121.2 (13.1)	125.2 (13.3)	129 (13.9)	0.001^a^
DBP (mmHg) Mean (SD)	72.7 (9.6)	75.8 (47)	71 (12)	74 (13.8)	0.5861^a^
Education level n (%)					<0.001^b^
Primary school	1 (0.9)	3 (2.8)	1 (0.9)	0 (0.0)	
High school	109 (99.1)	97 (90.7)	93 (91.2)	2 (1.9)	
University	0 (0.0)	7 (6.5)	8 (7.9)	104 (98.1)	
Working year n (%)					<0.001^b^
<5	74 (67.3)	38 (35.5)	81 (79.4)	74 (69.8)	
≥5	36 (32.7)	69 (64.5)	21 (20.6)	32 (30.2)	
Smoking status n (%)					<0.001^b^
Never	9 (8.1)	17 (15.8)	50 (49.0)	74 (69.8)	
Current	87 (79.0)	76 (71.0)	41 (40.1)	28 (26.4)	
Passive smoking at workplace	96 (87.2)	88 (82.2)	80 (78.4)	43 (40.5)	<0.001^b^
Alcohol consumption n (%)					<0.001^b^
Yes	95 (86.4)	89 (83.2)	60 (58.8)	66 (62.3)	
No	15 (13.6)	18 (16.8)	42 (41.2)	40 (37.7)	
Physical activity n (%)					<0.001^b^
Yes	53 (48.2)	27 (25.2)	12 (11.8)	56 (52.8)	
No	57 (51.8)	80 (74.8)	90 (88.2)	50 (47.2)	
Personal medical history n (%)					0.060^b^
Asthma	4 (3.6)	6 (5.6)	3 (2.9)	9 (8.5)	
Pneumonia	2 (1.8)	2 (1.8)	2 (2.0)	0 (0.0)	
Hypertension	0 (0.0)	0 (0.0)	0 (0.0)	2 (1.9)	
Others	1 (1.0)	1 (1.0)	4 (3.9)	7 (6.6)	
Never	103 (93.6)	98 (91.6)	93 (91.2)	88 (83)	
FEV_1_ Mean (SD)	2.7 9 (0.4)	2.64 (0.5)	2.71 (0.5)	2.80 (0.6)	0.14^a^
FEV_1_% of predicted Mean (SD)	75.8 (12.1)	76 (14)	76.7 (14.8)	80.6 (11.3)	0.076^a^
FVC Mean (SD)	3.02 (0.71)	3.34 (0.53)	3.44 (0.78)	2.85 (0.48)	<0.001^a^
FVC% of predicted Mean (SD)	75.2 (13.1)	81.6 (10.6)	82.8 (11.8)	80.6 (11.3)	<0.001^a^
FEV_1_/FVC Mean (SD)	78.5 (11.8)	77.6 (13.3)	79.4 (13)	79.88 (8.3)	0.51^a^

*%* percent, *SD* standard deviation, *FEV*_*1*_ Forced expiratory volume in the first second, *FVC* Forced vital capacity, *FEV*_*1*_ */FVC* Ratio of Forced expiratory volume in the first second and Forced vital capacity, *kg/m* ^*2*^ kg per square meter, a, Anova, b, Chi square

**Fig. 2:**
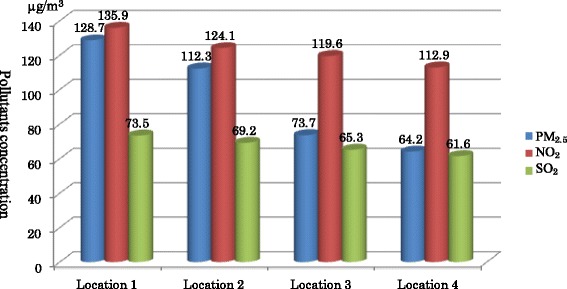
Pollutants concentration in different locations. Particulate matter 2.5 (PM_2.5_ μg/m^3^), Nitrogen dioxide (NO_2_ μg/m^3^), Sulfur dioxide (SO_2_ μg/m^3^), Microgram per cubic meter (μg/m^3^)

Concentration of PM_2.5_ was: 128.7 ± 3.40 μg/m^3^, 112.3 ± 4.43 μg/m^3^, 73.7 μg/m^3^ ± 3.13, and 64.2 μg/m^3^ ± 2.01 in location1, 2, 3, and 4 respectively. Whereas NO_2_ concentration was: 135.9 ± 2.16 μg/m^3^, 124.1 ± 6.73 μg/m^3^, 119.6 ± 2.55 μg/m^3^, and 112.9 ± 2.96 μg/m^3^, in location1, 2, 3 and, 4 respectively (Fig. 2).

Prevalence of respiratory symptoms was statistically significant in the study groups with *p* < 0.001, except for the morning cough. For impaired pulmonary function, 21.9% of bus conductors, 10.9% of bus drivers, 15.4% of taxi motorcyclists and 7.1% of high school teachers had mixed syndrome with *p* < 0.05 reported in the Table 2.

**Table 2:** Prevalence of respiratory symptoms in the study population

Symptoms *n* (%)	Bus conductors *n* = 110	Bus drivers *n* = 107	Taxi motorcyclists *n* = 102	High school teachers *n* = 106	*p* value
Cough					
Morning cough	11 (10)	13 (12.1)	9 (8.8)	5 (4.7)	0.281
At least 4 days a week	49 (44.5)	45 (24)	36 (35.2)	15 (14.1)	<0.001
Phlegm					
Morning phlegm	26 (23.6)	19 (17.7)	24 (23.5)	5 (4.7)	<0.001
At least 4 days a week	45 (40.9)	35 (32.7)	27 (26.4)	9 (8.4)	<0.001
Wheezing > 16y old	25 (22.7)	5 (4.6)	7 (6.8)	8 (7.5)	<0.001
Short breath	30 (27.2)	38 (35.5)	43 (42.1)	17 (16)	<0.001
Chest pain	19 (17.2)	0 (0.0)	0 (0.0)	14 (13.2)	<0.001
Restrictive syndrome	26 (27.3)	22 (23.2)	29 (30.4)	14 (14.7)	0.061
Mixed syndrome	16 (21.9)	8 (10.9)	10 (15.4)	6 (7.1)	0.042
Obstructive syndrome	7 (10.9)	6 (9.1)	6 (9.8)	3 (3.7)	0.372

Chi square test, *y* year, *%* percent, *p* < 0.05

Risk of impaired pulmonary function related to working year or duration of exposure was OR 4 (95% CI 2.11, 7.58) for bus conductors, OR 4.37 (95% CI 2.20, 8.69) for bus drivers, OR 4.53 (95% CI 2.35, 8.72) for taxi motorcyclists, OR 2.63 (95% CI 1.91, 6.90) for high school teachers (results not shown).

Morning cough was associated with bus conductors occupation, aOR 2.94 (95% CI 1.30, 6.64) and bus drivers, aOR 2.39 (95% CI 1.04, 5.48). In addition, a significant association with mixed syndrome, aOR 7.64 (95% CI 1.83, 31.67) was found for bus conductors. Whereas, restrictive syndrome, aOR 3.22 (95% CI 1.35, 7.69) was associated with taxi motorcyclists occupation (Table 3).

**Table 3:** Multivariate analysis of association between occupation groups, respiratory symptoms and respiratory impaired function

Respiratory symptoms	Bus Conductors *n* = 110 aOR 95% CI	Bus drivers *n* = 107 aOR 95% CI	Taxi motorcyclists *n* = 102 aOR 95% CI	High school teachers *n* = 106 aOR 95% CI
Cough				
Morning cough	2.94 (1.30, 6.64)^**^	2.39 (1.04, 5.48)^*^	2.01 (0.92, 4.40)	1.00
At least 4 days a week	4.87 (2.51, 9.45)^**^	4.40 (2.25, 8.58)^*^	3.30 (1.67, 6.53)^*^	1.00
Phlegm				
Morning phlegm	4.66 (1.70, 12.79)^**^	2.51 (0.88, 7.15)	3.52 (1.30, 9.54)^*^	1.00
At least 4 days a week	7.46 (3.41, 16.30)^**^	5.23 (2.36, 11.58)^*^	3.8 (1.72, 8.74)^*^	1.00
Wheezing > 16 y-old	1.35 (0.46, 3.97)	0.23 (0.60, 0.91)^*^	0.40 (0.12, 1.33)	1.00
Short breath	0.88 (0.38, 2.06)	1.05 (0.45, 2.42)	1.66 (0.79, 3.50)	1.00
Chest pain	1.49 (0.41,5.42)	–	–	1.00
Impaired pulmonary function				
Restrictive syndrome	3.13 (1.25, 7.80)^*^	1.43 (0.55, 3.75)	3.22 (1.35, 7.69)^*^	1.00
Mixed Syndrome	7.64 (1.83, 31.67)^*^	1.85 (0.42, 8.09)	2.61 (0.67, 10.08)	1.00
Obstructive Syndrome	4.49 (0.70, 28.56)	1.49 (0.23, 9.57)	2.90 (0.56, 14.97)	1.00

*aOR* adjusted odds ratio for Age, BMI, working year, smoking status, alcohol consumption, physical activity, personal respiratory history, *CI* confidence interval, *y*, year, ^*^ *p* < 0.05, ^**^ *p* < 0.01

## Discussion

Few studies have examined the potential role of RTE as a risk factor of impaired pulmonary function among the transit workers, particularly bus conductors.

The present research has assessed the relationship between occupation exposed to road-traffic emissions and respiratory health of Congolese bus conductors, bus drivers, and taxi-motorcyclists in Kinshasa.

To our knowledge, this study is the first to evaluate the respiratory health of these exposed groups in DRC.

After adjusting for a number of potential confounders, findings are depicted as follow:

1. Air pollutants measurements (PM_2.5_, NO_2_, and SO_2_) in outdoor, in bus, in classroom were higher than the normal range of World Health Organization Guidelines.
2. The prevalence of respiratory symptoms was higher in the exposed groups (bus conductors, bus drivers, taxi motorcyclists) than the control group (high school teachers).
3. Exposed groups had a decreased lung function and greater at risk to impaired pulmonary function than control group.
4. Exposed groups who worked for five years or more were at greater risk to impaired pulmonary function and they presented more pulmonary symptoms than the control group.

Our study reports that the level of outdoor PM_2.5_, NO_2_, and SO_2_ in different selected locations was higher than the normal range of the WHO guidelines. Thus, the maximum concentration of PM_2.5_ found was 134 μg/m^3^ and the minimum concentration was 62 μg/m^3^. Furthermore, the average concentration of PM_2.5_ was 94.72 ± 27.49 μg/m^3^ in selected locations. This value is close to the concentrations above 100 μg/m^3^ in Kampala, Uganda and 128.04 μg/m^3^ in Delta Niger, Nigeria [12, 20]. Although the methods of assessment were different; Uganda’s study used the permanent station monitor whereas our study the fact of limiting the financial support and Nigeria’s study used portable gas monitor. That DRC, Uganda and Nigeria are developing countries with lack of good policies in environment management could explain these similar results.

Our study found that bus conductors and bus drivers were more exposed to particulate matter than high school teachers (results not shown).

Similar results were reported by Krzyzanowski M et al., in Copenhagen, and two other studies in Taipei and in Madrid where the people use motorcycles on road, bus/mass rapid transit commuters walk or wait along commuting routes have exhibited to high personal particulate matter exposure due to the traffic-volume and environmental factors contributed in the high personal particulate matter exposure for people use motorcycles [1, 21, 22].

In our study the prevalence of almost all respiratory symptoms was significantly higher in the exposed group than the control group. Similar results were reported by Zuskin et al., comparing drivers, mechanics compared with office workers [23].

Exposure to RTE increased the burden of environment and induced respiratory symptoms in exposed groups than the control groups. Same results were reported at different places around the world by Schwander S et al., in Kampala, by Zhou et al., in Shanghai, by Karita K et al., in Bangkok and by Estevez-Gracia JA et al., in Bogota [3, 24–26]. The same trend was reported by Brant TC in Saopaulo, even in the general population and no-smoking commercial motorcyclists [27].

In contrast: Studying the prevalence of respiratory symptoms in 7,154 state road transport workers in India, Monica B et al., found that the prevalence of respiratory symptoms were significantly higher in office workers (34.9%) as compared to drivers (24.2%), conductors (25.4%), and 30.0% in garage workers [28]. Also, Biqert C et al. reported that a short- term exposure to fractional exhaled nitric oxide did not affect the pathway of the exhibited subway workers airway or their lung function [29].

Type of location, characteristics of population, method used for measurement, time of exposure, the composition and concentration of ambient pollutants, susceptibility of given population to oxidative stress, workplace condition could explain the different results in the similar epidemiological studies.

Present research has shown that the prevalence of mixed syndrome was 21.9% for bus conductors and 10.9% for bus drivers and restrictive syndrome was 27.3% for bus conductors, 23.2% for bus drivers. These results are close to the study conducted by Chattopadhyay BP et al., who found that the impaired pulmonary function was associated with exposure to automobile exhaust, especially, 30.4% at risk of developing the restrictive syndrome for conductors and 28.9% for drivers [30]. Similar results in the decrease of pulmonary function associated to traffic-related air pollution were reported in Mexican, Indian and in Danish studies [31–33].

Our study has reported that, after adjusting for confounding factors, occupation exposed to RTE were associated with mixed syndrome and restrictive syndrome, especially among bus conductors and taxi motorcyclists. Bus conductors were seven times at risk to develop mixed syndrome with aOR: 7.64 (95% CI 1.83, 31.67), whereas taxi motorcyclists were three times at risk of developing restrictive syndrome with aOR: 3.22 (95% CI 1.35, 7.69). Indeed, the Congolese policy in the transportation sector requires maintenance time be respected, the wearing of a crash helmet by motorcyclists also, prohibits bus conductors to be outside the bus but the applicability of those measures is not effective, this make transit workers in Kinshasa more vulnerable to the impaired respiratory function. Framingham heart study reported the close results that the decline of lung function was associated with a long term exposure to traffic and PM_2.5_ at relatively low levels concentration (μg/m^3^) [10]. Additionally, our research shows that occupation exposed groups who worked for five years or more have presented four times at risk of developing impaired pulmonary function. High school teachers group had more than twice at risk of developing impaired pulmonary function. Similar results were found in Nigerian transit workers, worked for five years [12]. Whereas, Croatian study has found that this association was significant among exhibited bus drivers and mechanics employed for more than 10 years [23].

Otherwise, the fact that the most of developing countries like DRC, the high school teachers use continuously a piece of chalk with the black board and inhale the dust releases by this process, is a plausible explanation of impaired respiratory function in this group. Little is known on the exact mechanism of impaired respiratory function according to exposure to traffic-related air pollution [27]. Our study as several others would support the assumption of part of oxidative stress and inflammation in the alteration of respiratory function and partial obstruction of pathway [34, 35].

### Strengths and limitations

This is the first study elucidated the association between occupation groups of transit workers exposed to RTE and impaired respiratory function in the Democratic Republic of the Congo, using the American Thoracic Society respiratory symptoms questionnaire and spirometry. Also the present study has assessed traffic volume and air pollutants measurements (PM_2.5_, NO_2_ and SO_2_) outdoor, in the bus, in the classroom.

Despite these strengths, the study has some limitations;

First, a cross-sectional design that could not establish the causal relationship between the exposed groups and the occurrence of pulmonary function decline.

Second, the measurements of pollutants were carried out at selected locations limiting the generalization of the results to other geographic locations with very different engine, fuels used, topography and workplace condition.

Third, the pollutants (PM_2.5_, NO_2_, and SO_2_) were measured by a portable gas monitor. The use of a spatial-temporal land model, instead of portable gas monitor would have provided more accurate information on each participant. Finally, no data on residential history of the participant or on the time spent outside were collected.

## Conclusions

As conclusions, the present study found an association between occupation groups of transit workers exposed to RTE and impaired pulmonary function, with a greatest risk of mixed syndrome among bus conductors and a risk of restrictive syndrome among both bus conductors and taxi motorcyclists. Also, prevalence of impaired pulmonary function increased with working length. Hence the imperative needs to regularize this category of profession and apply important preventive policy. Follow up study is needed to establish the evidence of causal relationship.
